# Three-to-one analog signal modulation with a single back-bias-controlled reconfigurable transistor

**DOI:** 10.1038/s41467-022-34533-w

**Published:** 2022-11-17

**Authors:** Maik Simon, Halid Mulaosmanovic, Violetta Sessi, Maximilian Drescher, Niladri Bhattacharjee, Stefan Slesazeck, Maciej Wiatr, Thomas Mikolajick, Jens Trommer

**Affiliations:** 1 NaMLab gGmbH, Noethnitzer Strasse 64a, 01187 Dresden, Germany; 2 GlobalFoundries Fab 1 LLC & Co. KG, Wilschdorfer Landstraße 101, 01109 Dresden, Germany; 3 TU Dresden, Chair for Nanoelectronics, Noethnitzer Strasse 64, 01187 Dresden, Germany

**Keywords:** Electrical and electronic engineering, Electronic devices, Electronic devices, Electronic properties and materials, Electronic and spintronic devices

## Abstract

Reconfigurable field effect transistors are an emerging class of electronic devices, which exploit a structure with multiple independent gates to selectively adjust the charge carrier transport. Here, we propose a new device variant, where not only p-type and n-type operation modes, but also an ambipolar mode can be selected solely by adjusting a single program voltage. It is demonstrated how the unique device reconfigurability of the new variant can be exploited for analog circuit design. The non-linearity of the ambipolar mode can be used for frequency doubling without the generation of additional harmonics. Further, phase shifter and follower circuits are enabled by the n- and p-type modes, respectively. All three functions can be combined to create a 3-to-1 reconfigurable analog signal modulation circuit on a single device enabling wireless communication schemes. Both, the concept as well as the application have been experimentally demonstrated on industrial-scale fully-depleted SOI platform. The special transport physics in those structures has been analyzed by TCAD simulations as well as temperature dependent measurements.

## Abstract (web summary)

Designing efficient reconfigurable field effect transistors remains a challenge. Here, the authors develop a transistor with three distinct operation modes, realized directly on an industrial 22nm FDSOI platform, demonstrating a reconfigurable analog circuit element with signal follower, phase shifter, and frequency doubler operation.

## Introduction

Advances in complementary metal-oxide-semiconductor (CMOS) industry are driven by the continuous shrinking of transistor features sizes, which is traditionally associated with increased operation frequencies, lower power consumption and decreased cost per unit. This trend, however, is impeded since the transition from bulk transistors to more complex technologies like FinFETs and silicon-on-insulator (SOI) devices results in approaching physical limitations and the need for cost-intensive processing technology. However, while the single thread performance and power dissipation are stagnating, the density of transistors on a chip is further growing exponentially. This is reasoned in a second electronic mega-trend, which is functional diversification^1^. Microprocessors are not anymore designed for the sole purpose of performing arithmetic and logic functions. For instance, embedded non-volatile memory functionality is intensively researched as an option to enable microprocessors to overcome the van Neumann bottleneck, by manipulating the data directly where it is stored^2,3^. Another example is the integration of analog functionalities into the digital system, like mmWave/radar processing needed for automotive application^4^. A further increase in functional density is expected from the co-integration of emerging devices, whose functionality goes beyond that of classical MOSFETs such as resonant tunnel diodes^5^ and single-electron transistors^6^.

A particularly promising group of devices providing such an added functionality are reconfigurable field effect transistors (RFETs). While the polarities in classical CMOS are defined mainly by impurity doping of the channel and contact regions^7^, RFETs employ electrostatic doping to control the carrier injection through Schottky barriers (SB) at source and drain^8–11^. As a result, the user can electrically select the device functionality to be p-type or n-type. This feature can be exploited for a variety of applications, from general digital circuit design^12–14^ over hardware security primitives^11,15,16^ to neuronal networks^17,18^. However, research in the field has been mainly focused on digital applications, neglecting the potential of device-level reconfiguration for analog signal processing. Here, particularly the non-linear characteristics of Schottky barrier FETs are of interest for frequency multiplication, an important property needed in communication systems^19^.

In this article, we propose a ew reconfigurable device variant that can be used for three-to-one analog signal processing. The devices are built on a modified industrial FDSOI platform proving full CMOS compatibility and scalability. The unique property of applied dynamic back-bias (BB) offered by the FDSOI technology is used for programming the device characteristics. In particular, p-type, n-type, and ambipolar device modes are used to demonstrate a signal follower, phase shifter, and frequency multiplier operation, respectively. Dynamic switching between the three modes solely by adjusting the voltage of a single independent electrode is demonstrated experimentally.

## Results

### Back-bias reconfigurable field effect transistor

Reconfigurable transistor operation is based on the ambipolar transport in nanoscale Schottky junctions, where the current is dominated by tunneling injection of either electrons or holes through the Schottky barrier. The current *I*_*ON*_ can be approximated by the Wenzel-Kramers-Brillouin (WKB) approximation^20^:

1 $${I}_{{{{{{\mathrm{on}}}}}}}=\exp \left(-\frac{4\lambda \sqrt{2{m}^{*}{\Phi }_{{{{{{\mathrm{SBH}}}}}}}}}{3\hslash }\right)$$

where *λ* is the geometric screening length, m* is the effective mass, and Φ_SBH_ the natural Schottky barrier height for electrons or holes, respectively. If the product m* ⋅ Φ_SBH_ is equally large for holes and electrons, a symmetric ambipolar operation between p-type branch and n-type branch can be achieved. Thus, reconfigurable FETs require metal/semiconductor contacts that align close to the mid-gap Fermi level (*E*_F_) of the channel material. For silicon devices this is the case with contacts made from NiSi_2_. This material also provides an epitaxial relation to silicon with low-lattice mismatch^21^.

In order to be applicable for complementary logic, the ambipolar operation must be controlled to yield p- or n-operation modes. Thereto, the injection of undesired carriers, i.e., the undesired branch of the ambipolar transfer characteristics, is suppressed by structures with multiple independent gates. Recent attempts include implementations with two^9^, three^22^, or even more frontgates^23^. Also buried gates^24^ or a mixture of front and backgates^11,25^ have been used. Independent of the actual layout, all of these device concepts have in common that they aim on a deliberate spacial separation of the control gates, which steer the device, and the polarity gates, which select the active carrier type. Most often, either electrons or holes are completely filtered, leading to a clear separation between the p- and n-type mode and very low OFF-currents *I*_OFF_ (see also Supplementary Table 1). However, the large multi-gate structures come at the cost of a high-area overhead, which hinders an effective co-integration with CMOS. Also, the properties of the underlying ambipolar operation mode are only accessible if several gates are tied together.

Both facts are distinctively different in the back-bias RFET variant conceived in this work. Instead of well separated independent gates, both, control gate as well as polarity gate, couple to the whole channel region. This is achieved by a thin planar channel, having a front and backgate both covering the whole channel. Naturally, this design consumes less space to be brought onto a chip. We will show that in addition to the p- and n-type modes the ambipolar characteristics, to be used for analog applications, are well preserved due to the altered band alignment.

**Fig. 1:**
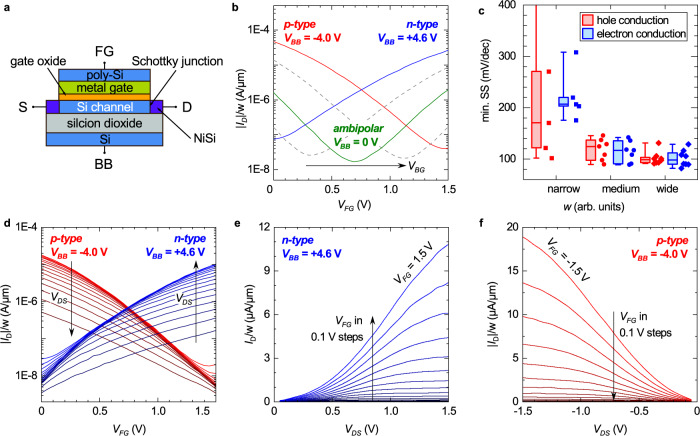
Back-bias reconfigurable field effect transistor (BB-RFET) integrated on 300 mm wafers using 22 nm FDSOI technology. **a** Schematic image of a single device with materials and contacts, comprising source (S), drain (D), front-gate (FG), and a back-bias (BB) contact. **b** Three distinct operation modes accessible by changing the applied back-bias (*V*_BB_). **c** Distribution of minimal subthreshold swing SS in ambipolar mode for *V*_DS_ = 0.1 V measured at devices with varying width as compared to Fig. 1b. Boxplot shows median with 25% and 75% quartile. Outliers are indicated. **d** Full families of transfer characteristics for n-type (blue) and p-type (red). *V*_DS_ is varied from 0.1 to 1.5 V in steps of 0.1 V. **e** Family of output characteristics in n-type operation. **f** Family of output characteristics in p-type operation. *w* is the channel width in all plots.

The concept has been demonstrated for FDSOI channels as shown by the schematic representation in Fig. 1a. A thin intrinsic Si channel on a 20 nm thick buried oxide (BOX) is used. A stack consisting of polysilicon/TiN (gate electrode), HfO_2_ (high-k dielectric), and SiO_2_ (interface layer) forms the frontgate (FG). At the source and drain contacts Ni is intruded by a rapid thermal annealing process to form metallic NiSi_x_/semiconductor junctions directly within the channel. The n-doped well under the BOX is contacted separately to form a second gate on the back-side of the device. Devices have been fabricated with various gate dimensions ranging from 70 nm to 2 μm in length and 160 nm to 2 μm in width, making them compatible with the i/o devices of the 22 nm FDSOI CMOS. All transistors have been fully integrated next to working MOSFETs on 300 mm wafers with complete back-end-of-line (see also Supplementary Fig. 1).

Key transfer characteristics of an exemplary device with 100 nm gate length and 250 nm width are shown in Fig. 1b. The device was tailored for an operation voltage *V*_DD_ of 1.5 V, i.e., both front-gate voltage *V*_FG_ and drain-source *V*_DS_ are ranging between 0 and 1.5 V. It can be seen that the ambipolar transfer curve of the underlying Schottky barrier FET is well preserved if a back-bias of zero volt is applied. The minimal voltage point *V*_MIN_ is centered at 0.70 V and relatively equal on-currents of 1.62E-7 A and 1.9E-7 A are achieved for the p- and n-branch, respectively. A high symmetry around *V*_MIN_ is imperative for the frequency doubling application explained later. Thus, in Fig. 1c the variability of minimal swing for both branches in the ambipolar mode at *V*_DS_ = +0.1 V are compared for different device sizes. Especially for medium and high channel width, which are the relevant ones for analog designs, hole and electron conductance show a very homogeneous distribution of slopes, which indicates a good p/n-symmetry. More narrow devices tend to show an increased swing and higher variability. Here, a further fine-tuning of the silicidation process is needed. Thus, we focused our analysis in this paper on the medium size and wide devices.

By application of a back-bias, the ambipolar transfer curve can be shifted seamlessly together with the voltage point *V*_MIN_. Figure 1b shows how for a sufficiently large positive back-bias voltage *V*_BB_ the falling branch is shifted out of range of the front-gate voltage so that only the rising branch of the curve remains and the transistor shows the behavior of an n-type FET. Correspondingly, for sufficiently large negative *V*_BB_, only the falling branch of the curve remains, i.e., the transistor behaves like a p-type FET. Therefore, the transistor can be operated effectively in three distinctively different operation modes: ambipolar (green), n-type (blue) and p-type (red). Note that the transition between them is transition-free, as indicated by the dashed intermediate characteristics. For the p-type (n-type) operation, an applied back-bias *V*_BB_ = –4.0 V (*V*_BB_ = +4.6 V) was chosen such that *V*_MIN_ of the corresponding saturation curve is aligned to *V*_DD_ or *GND*, respectively. Simultaneously, the p- and n-type curve cross at *V*_DD_/2 = 0.75 V, which is desirable for digital operation. Note that it would also be possible to choose the *V*_BB_ values in favor of an even better alignment of the on-currents, instead. The full set of *I*_D_–*V*_FG_ characteristics of p- and n-type for various *V*_DS_ are given in Fig. 1d. In order to keep the terminal at which the carriers are injected on the same side, the source contact is biased to 0 V for n-configuration and 1.5 V for p-configuration. In full ON-state, currents of 18 (10) μA/μm and an ON/OFF ratio of 890 (350) for p-type (n-type) mode are achieved. The ON/OFF ratio is limited by a flattening of the subthreshold characteristics close to the OFF-state for high absolute ∣*V*_DS_∣. The flattening originates from the on-setting injection of the opposite carrier type in the ambipolar curve. Minimal subthreshold swings of 300 mV/dec (308 mV/dec) in p- (n-) configuration are achieved for *V*_DS_ = 0.6 V. Threshold voltages for a current criterion of 100 nA/μm are 0.42 V and 0.33 V for p- and n-configuration, respectively. Note that higher ON/OFF ratios can be achieved by choosing higher values of ∣*V*_BB_∣ so that the current minimum is further shifted out of the front voltage range and coincidentally resulting in higher ON-currents.

In Fig. 1e, f, the recorded output characteristics are presented for n- and p-characteristics for a *V*_DS_ voltage range between 0 and 1.5 V in steps of 0.1 V. The FET-typical saturation of currents sets on for high absolute values of ∣*V*_DS_∣. For low ∣*V*_DS_∣, the curves follow a sublinear shape, which is a signpost behavior of FETs with Schottky barrier contacts^20^. The behavior is caused by the drain-sided Schottky barrier that limits the current additionally to the source-sided Schottky barrier as long as potential at the drain is not sufficiently pulled below the Fermi level of the source side by ∣*V*_DS_∣. At a first glance, the performance of the presented device has some natural limitations because of the concept of using a Schottky barrier contact. However, please note that this technology is not planned to completely replace classic CMOS devices, but rather provide an add-on functionality, where beneficial. Still, there are several pathways to explore in order to yield increased performance. Naturally, performance metrics can be improved with further scaling of channel length and gate oxide thickness^26^. Further, low-bandgap channel materials such as Ge or SiGe, which is already established for p-type transistors in CMOS, can be employed to lower *V*_TH_ and increase the drive currents and gain values. Also note that the analyzed devices have a quite equal width/length ratio, while for analog applications typically ultra-wide multi-finger devices are used. Finally, it is also possible to transfer the device variant to channel materials featuring a smaller screening length like semi-metallic graphene^27^ layered 2D materials, such as black-phosphorus and *WSe*_2_, as channel materials^10,11^ once the CMOS co-integration barrier of those materials has been lowered^28^.

### Transport analysis

**Fig. 2:**
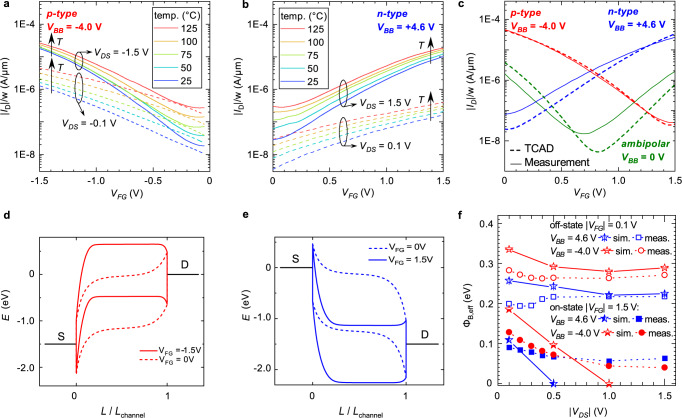
Analysis of back-bias RFET device physics. **a**, **b** p-type and n-type transfer characteristics in the temperature range from 25 to 125 °C, respectively. **c** Fitting of the measured transfer characteristics by TCAD simulations using a process simulation approach. **d**, **e** Band diagrams for a ∣*V*_DS_∣ of 1.5 V for p-type (red, *V*_BB _= –4.0 V) and n-type (blue, *V*_BB_ = 4.6 V) program, respectively. Solid lines represent ON-states, dashed lines represent OFF-states. Potential along the channel is given relative to the overall channel length *L*. **f** Extracted effective barrier values from both TCAD simulations and temperature-dependent measurements in the on- and off-states, respectively. A fixed tunneling distance *d*_eff_ of 2.8 nm was used to yield simulation results. Both methods agree qualitatively good. Lines are guides to the eye.

In order to yield more insights into the transport physics of the new device variant, we performed temperature-dependent measurements (Fig. 2a, b) and extracted the barrier properties of our transistors. A TCAD process simulation of our device has been set up and fitted to the experimental data (Fig. 2c). Effective barrier values have been calculated from the simulated band diagrams in the on- and off-states (Fig. 2d, e) using a simple tunneling distance model and are compared to the barrier values extracted from the experiment (Fig. 2f).

As it can be seen in Fig. 2a, b, the device exhibits a stable temperature behavior in the range from 25 to 125 °C. Other than classical MOSFETs having an inversion channel, both ON-current and OFF-current increase with temperature. This is reasoned in the increased injection of charge carriers over the Schottky barrier in the ON-state, which overshadows the effect of increased phonon scattering typically observed in MOSFETs. From the temperature data an effective barrier height *ϕ*_B,eff_ can be derived, which equals a thermal activation energy of the system^20^. If all external voltages converge towards zero, *ϕ*_B,eff_ is expected to approach the natural Schottky barrier height Φ_SBH_ for the respective carrier type. The resulting barrier values extracted from an Arrhenius plot of $\ln ({I}_{{{{{{\mathrm{D}}}}}}}/{T}^{2})$ versus 1/*T* as a function of *V*_BB_ and *V*_DS_ are shown in Fig. 2f. Note that we worked under the assumption that the thermal velocity as well as the density of states of the devices are rather independent of temperature in the elaborated temperature range^29^. We focused the analysis towards four distinct states, the ON- and OFF-states for p- and n-type mode, respectively. Unsurprisingly, for both modes the effective barrier is larger in the subthreshold region than in the respective saturation region. At ∣*V*_GS_∣ = 1.5 V, a small *V*_DS_-dependent barrier is present. Interestingly, the barrier does not vanish even at ∣*V*_DS_∣ = 1.5 V, indicating that higher ON-currents could be achieved by a more optimized device design, e.g., a better coupling of the front-gate or a channel material with a smaller bandgap^30^. The increase of *ϕ*_B,eff_ towards lower *V*_DS_ shows the build-up of a the drain-sided Schottky barrier, which limits the ejection of the current^31^. This matches nicely with the observed typical Schottky-type sublinear shape of the output characteristics visible for low *V*_DS_ in Fig. 1e, f. In the OFF-state, the energy barrier is mostly independent from *V*_DS_. Interestingly, *ϕ*_B,eff_ for p- and n-mode only sum up to 0.5 eV, which is roughly half of the bandgap *E*_*g*_. This is different to other RFET concepts and reasoned in the competing blocking potential between front-gate voltage and back-gate voltage. If *V*_BB_ would also be reduced towards 0 V also the barrier property would be expected to converge towards Φ_SBH_ again. The trend of the measured barrier values (circles and squares) agrees well with the predicted effective barrier height, assuming an effective tunneling distance *d*_eff_ of 2.8 nm, as extracted from the band diagrams yielded by the TCAD simulations (stars). Details on the approach are given in Supplementary Fig. 3 in the supplementary information.

### Frequency multiplication

Non-linear circuit elements can be used to generate an analog output signal, whose frequency is a multiple (harmonic) of its input frequency^19^. This functionality is of great interest in a variety of analog applications such as frequency mixers^32,33^, amplifiers and modulators^34^. A key challenge is to provide such frequency multiplication in a way that most of the energy is confined in the target frequency as often additional unwanted harmonics are generated^5^. Such harmonic generation leads to low conversion efficiencies. While it is possible to compensate for those effects with additional circuitry, this would largely increase area and power overheads. One approach to avoid higher order generation is the exploitation of symmetric device characteristics^5^. Given a perfect parabolic relation between the output and input of the device:

2 $${V}_{{{{{{\mathrm{OUT}}}}}}}=A+B{({V}_{{{{{{\mathrm{IN}}}}}}}-{V}_{{{{{{\mathrm{MIN}}}}}}})}^{2}$$

with A and B being constant parameters, and a sinusoidal input wave *V*_IN_ of frequency *f* having the form

3 $${V}_{{{{{{\mathrm{IN}}}}}}}={V}_{{{{{{\mathrm{MIN}}}}}}}+\frac{1}{2}{V}_{{{{{{\mathrm{DD}}}}}}}\sin (2\pi ft),$$

an output voltage with the following form can be derived

4 $${V}_{{{{{{\mathrm{OUT}}}}}}}=A+\frac{({{{{{\mathrm{B}}}}}}{{{{{{\mathrm{V}}}}}}}_{{{{{{\mathrm{DD}}}}}}}^{2})}{8}-\frac{({{{{{\mathrm{B}}}}}}{{{{{{\mathrm{V}}}}}}}_{{{{{{\mathrm{DD}}}}}}}^{2})}{8}\cdot \cos (4\pi ft).$$

From there it follows that perfect parabolic transfer characteristics lead to a perfect doubling of the frequencies without the generation of higher order harmonics^35^. Consequently, devices providing a symmetric parabolic output have been studied intensively, including Schottky FETs^36^, resonant tunnel diodes^5^, graphene FETs^32,37^, carbon nanotube (CNT) FETs^35^, and ferroelectric (FE) FETs^33,38^. Especially semi-metallic graphene devices have been proposed as promising material for frequency doubling, due to their high-current throughput and large achievable gains^34,39,40^. However, they need very high gate voltages, are limited with respect to the on/off ratio due to the missing bandgap, and CMOS co-integration is still not solved^28^. Moreover, frequency doubling has been demonstrated in principle, but a sophisticated solution to adaptively tune the operation point *V*_MIN_ is missing. In most cases, the carrier wave and the input signal are superimposed at the gate^33,39^. Therefore, *V*_MIN_ has to be adjusted by carefully tuning the fabrication process, instead. For example, the usually unwanted gate-induced-drain-leakage (GIDL) is engineered to achieve parabolic transfer characteristics in FE-FETs^38^. As opposed to all those technologies, the bias point *V*_MIN_ in BB-RFETs is not fixed by the technology. Instead, the ambipolar transfer curve can be seamlessly shifted by the applied back-bias. Thus, the working point of frequency doubling can be adapted to the DC-offset of the input signal (see also Supplementary Fig. 2). It is conceivable that an adaptive use of the back-bias as proposed for digital designs, can be established to largely improve conversion efficiencies^41^.

**Fig. 3:**
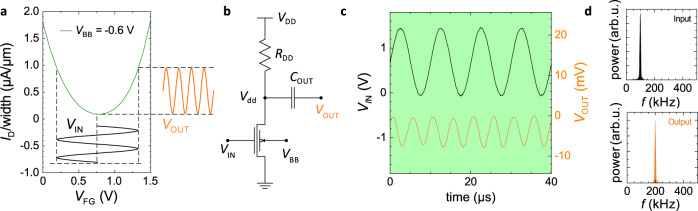
Frequency doubling utilizing the parabolic transfer characteristic in ambipolar mode. **a** Ambipolar transfer characteristics of the used BB-RFET when steered by *V*_BB_ = –0.6 V at the backgate and a drain-source voltage of *V*_DS_ = 1.5 V(green). Drawn-in sine curves schematically symbolize the input and output signals at the operation point. **b** Circuit diagram of the single transistor setup used for frequency manipulation. The output *V*_OUT_ is capacitively coupled to the *V*_dd_ node tapped between a 3.3 kΩ load resistor *R*_DD_ at the supply potential and the BB-RFET. The sinusoidal input *V*_IN_ is applied at the frontgate, while the back-bias voltage *V*_BB_ determines the manipulation mode of the circuit. **c** Measured input (black, left ordinate) and output (orange, right ordinate) signal of the setup over time. Here, *V*_DD_ of 1.75 V and *V*_BB_ of –0.4 V are applied. **d** Frequency spectra of the input and output signals plotted in **c** calculated by fast-Fourier transform (FFT).

In this study, we usually aimed for centering *V*_MIN_ around *V*_DD_/2. Figure 3a shows that the near perfect parabolic shape of the transfer characteristics is achieved when a back-bias *V*_BB_ = –0.6 V is applied. Fitting the curve with an ideal parabola yields an R-Square value of 0.989. By superimposing a sinusoidal input signal with frequency *f*_0_ at the frontgate, which is biased to *V*_MIN_, the drain current will output a signal whose fundamental frequency is 2 ⋅ *f*_0_. Owing to the non-linearity, both positive and negative input half cycles will result in positive drain current half cycles, so that each half cycle of the input signal will lead to a full cycle at the output. The behavior follows out of equation (4) and is depicted schematically by the insets in Fig. 3a. The frequency doubling is experimentally proven using a setup employing a single BB-RFET as shown in Fig. 3b. Here, a device with 2 μm width and 90 nm gate length is used for demonstration. One S/D terminal of the transistor has been grounded while the other S/D terminal is connected via a 3.3 kΩ resistor to the supply voltage. Meanwhile, the output is measured behind a dc-decoupling capacitor of 100 nF.

In Fig. 3c, the frequency doubling is clearly visible. For a sinusoidal input (left axis) with 0.75 V for both amplitude and dc-offset and a frequency of 100 kHz, the output (right axis) results in a cosine with 200 kHz. Spectral analysis via fast-fourier-transform (FFT) confirms that the majority of the output signal power is confined to 200 kHz. Higher order harmonics are nicely suppressed having a peak power of 2% or less as compared to the target harmonic. Frequency multiplication was also tested for other input frequencies in the range from 10 to 100 kHz. For frequencies exceeding 1 MHz the analysis is limited by setup parasitics and the resolution of the used measurement hardware (see also Supplementary Fig. 5).

### Reversible phase shifter

**Fig. 4:**
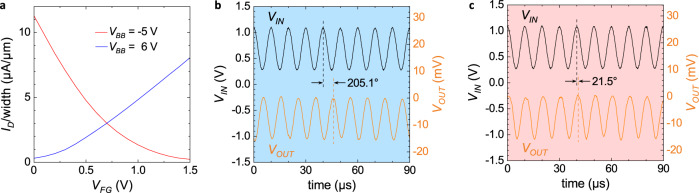
Demonstration of phase shifting and following modes, with the same BB-RFET and setup as shown in Fig. 3b. **a** Individual transfer characteristics showing the underlying p-type (red) and n-type (blue) behavior for varied back-bias voltages *V*_BB_. **b** When the BB-RFET operates in n-configuration, the output signal *V*_OUT_ (orange) is delayed by 205.1°. **c** When the BB-RFET is steered to p-configuration, the circuit is in the follower mode so that the output signal is equal to the input signal in frequency and phase except for some delay of 21.5°. This is a 183.6° phase shift compared to the mode in **b**.

The same setup as shown in Fig. 3b can also be used as a digital phase modulator^42^. Here, instead of the ambipolar mode, the p- and n-modes of the device are utilized. If the RFET is put into the n-type program at *V*_BB_ = 6 V (see Fig. 4a), a rudimentary NMOS-inverter-like behavior is achieved. In case of a sine as input signal at the gate, the result is an inversion of the sine or in other words a 180° phase shift. We will therefore call this operation mode shifter in the following. The operation is experimentally verified for an input frequency of 100 kHz in Fig. 4b. Data for higher frequencies can be found in Supplementary Fig. 6 in the supplementary information. In general, an ideal sine-shaped output signal can be achieved when the *g*_*m*_ of the transfer curve is almost constant over the input signal voltage range. For this analysis we have therefore limited the input signal amplitude to 0.4 V around a dc-offset of 0.75 V. Nevertheless, it should also be noted that a further shifting of the transfer curve towards lower voltages by means of the back-bias will move a more linear segment of the transfer curve into the voltage range of the frontgate.

When the transistor operation is changed to p-type behavior, the output voltage is not inverted anymore and the same setup turns into an input phase follower circuit. To access the p-operation, the back-bias voltage has to be changed to –5 V. Figure 4c shows the resulting output voltage behind the capacitor for a frequency of 100 kHz and an input signal amplitude of 0.4 V with a dc-offset of 0.75 V. The phase has changed by 183.6° compared to Fig. 4b and now almost follows the input signal, except for a delay of 21.5° due to the capacitive load.

Naturally, the applied back-bias voltage allows to reversibly switch between the phase shifter and follower operation modes. It should be noted that both modes only transport the fundamental wave to the output. This is because the non-linear behavior around the bias point *V*_MIN_ is outside of the operation range of the input signal. This indicates that also the frequency doubling mode can be turned on and off at run-time, which we will prove in the following.

### Three-in-one signal modulation

**Fig. 5:**
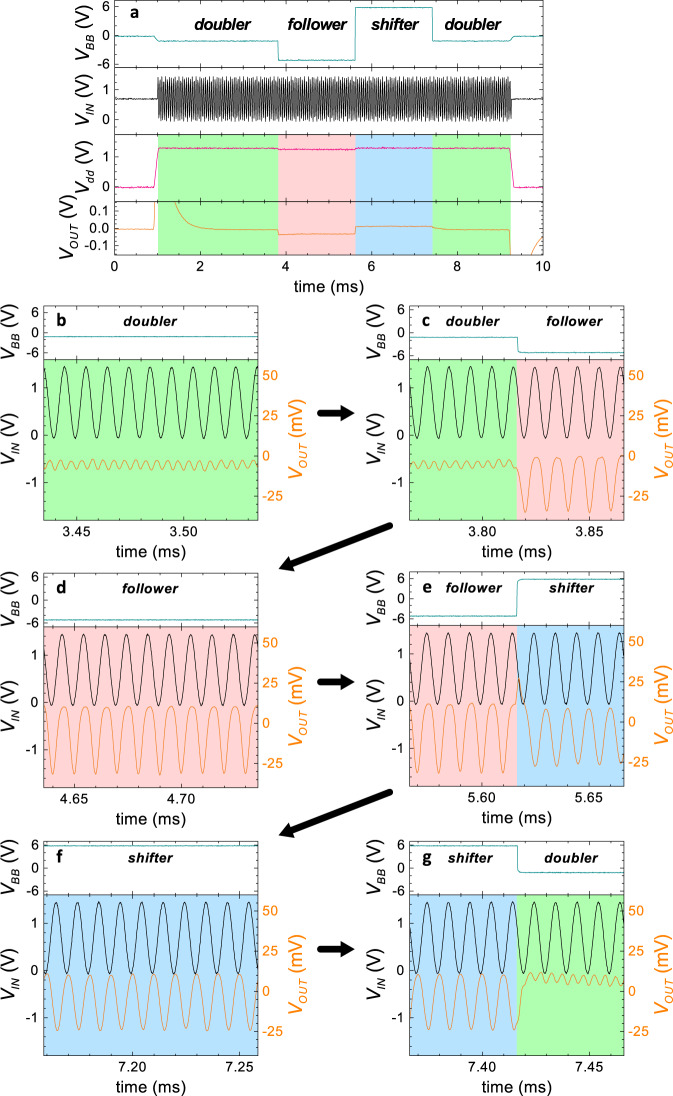
Embedded 3-to-1 signal modulation measured with the same setup and BB-RFET shown in Fig. 3b. A single device can be dynamically reconfigured between follower (red shade), phase shifter (blue shade) and frequency doubler (green shade) by the applied voltage at the back-gate *V*_BB_. **a** Large-scale overview of the applied and measured voltages for the complete reconfiguration sequence measured at 100 kHz. **b**–**g** 100 μs long sections of the signals for the individual operation modes or moments of switching. The back-bias voltage is plotted in the upper plots and the input (left axis) and output voltage (right axis) in the lower plots. Arrows indicate the chronological order of the plots. Please note that the DC component in the output waveforms is due to the transitory effect of the measurement.

Ultimately, we demonstrate in Fig. 5 that the single transistor setup can be also reversibly and rapidly switched between follower, phase shifter and frequency doubler operation modes at run-time. For this sake the back-bias voltage is steered consecutively to –1.0 V, –5 V, –6 V, and –1.0 V so that a seamless transition between all three operation modes as shown in Fig. 5a is achieved. The measurement was carried out using the setup and transistor from Fig. 3b, using *V*_DD_ of 1.5 V and an input frequency of 100 kHz. Figure 5b–g display zoomed in images at the crucial operation points. At the beginning and end of the measurement, the device is set to the ambipolar mode and the output frequency is doubled to 200 kHz (Fig. 5b). After switching to a negative *V*_BB_, an instantaneous transition to the follower mode is shown in 5c. It can be seen that an additional dc-offset is present until charging the capacitive load after switching. Figure 5d presents the steady-state operation in the follower mode for which the output phase almost equals the input phase. In Fig. 5e the mirroring of the output signal in the moment of switching between follower and phase shifter mode is shown. The phase shifter mode is then depicted at steady back-bias in Fig. 5f. Finally, a reconfiguration back to the frequency doubler mode is achieved, proving the reversibility of the configuration (Fig. 5g). Thus, a dynamic reconfigurable 3-to-1 signal modulation is possible with our back-bias RFET base structure.

## Discussion

The proposed setup can be used to emulate a variety of modulation schemes as needed for wireless communication systems. Two basic concepts that can be derived from our data are binary phase shift keying (BPSK) or binary frequency shift keying (BFSK). Details on both schemes are given in Supplementary Fig. 7 in the supplementary information. Here, digital data (1s or 0s) applied at the back-gate are used to modify either phase or frequency of a carrier signal applied at the front-gate *V*_IN_. This way the data bit stream is used to shift the bias point *V*_MIN_ of the *I*_D_–*V*_G_ curve. In case of the BPSK the back-bias is switched in the whole voltage range between *V*_BB,p_ and *V*_BB,n_. In case of BFSK only half of the voltage range is required. Beyond those simple schemes, it can be conceived that the back-bias RFET concept can also be applied for more sophisticated frequency processing applications, like frequency mixing^32^. The main benefit for our technology is that a frequency doubling can be achieved with a suppression of additional harmonics and without the need for inductive elements, which are hard to integrate in a CMOS platform. Owing to this, a lot of area can be saved using the BB-RFET technology. For example a compact low-power frequency doubler in the very same base technology needs 33,128 μm^2^ of area^43^, while our core as proposed in Fig. 3b requires only 2.1 μm^2^. This massive gain comes at the trade-off of a substantial lower operation frequency. Also, no amplifiers or stabilizing circuits are considered. Application scenarios have to be found, where the benefits of the new technology out-weight the challenges. One specific area could be hardware security^11^ for analog systems, where the back-bias is operated as a key to set the functionality at the front side, nicely hiding the actual functionality in the design. We believe that our work will stimulate the design and application of reconfigurable field effect transistors, both due to the newly available platform based on a 22 nm FDSOI process, as well as the added functionality of our 3-to-1 analog signal modulation circuits.

In summary, we have demonstrated a back-bias reconfigurable transistor (BB-RFET) variant that can be switched between p-type, n-type and ambipolar modes solely by the chosen applied back-bias voltage. The physical mechanisms behind the different operation modes have been analyzed in detail by electrical measurements and simulations. The potential of the reconfigurability for analog circuit designs has been explored. In the ambipolar mode, the device exhibits nearly perfect parabolic transfer characteristics, which can be applied for frequency doubling without the generation of additional harmonics. Furthermore, p- and n-type modes enable a reversible switching between a signal follower and a 180° phase shifting mode. The modes can be combined to yield a 3-to-1 reconfigurable frequency modulation circuit on a single device enabling wireless communication schemes, such as BPSK and BFSK. Both, the device as well as the application have been experimentally demonstrated on an industrial-scale fully depleted SOI platform. By the unique device design of the BB-RFET, having front and back-gate coupling to the whole channel, the individual device size has been reduced compared to prior-art RFETs and enables a co-integration into scaled CMOS processes.

## Methods

### Device fabrication

The presented back-bias reconfigurable field effect transistors have been processed based on GlobalFoundries 22FDX™ platform^7^. The devices are built on thin virtually doping-free SOI substrates with 20 nm buried oxide thickness. The integration flow shares most modules with 22 nm n-FETs, such as shallow-trench isolation (STI), gate-first high-k metal gate (HKMG) front-gate integration, and spacer deposition. In order to allow for high gate voltages, an extended SiO_2_ gate oxide interface has been used. Modified source and drain terminals are applied to allow for a sufficient silicide intrusion into the channel in order to create silicide-to-semiconductor junctions. A post-STI hybrid etch process is used to form back-gate contacts^7^. Finally, the back-end-of-line (BEOL) connections are processed. The entire process requires no changes to the 22FDX™ design rules and no additional masks with critical dimensions.

### Electrical characterization

In all, 300 mm wafers have been characterized on an Accretec UF3000-e fully automatic probe station. The chuck temperature has been adjusted from 25 to 125 °C. Device characteristics have been collected using a Keithley 4200A Semiconductor Characterization System with four 4210-SMU source measurement units. The sinusoidal waveforms have been supplied by an arbitrary waveform generator (Agilent 81160A). Analog output waveforms of *V*_OUT_ were collected using an Agilent DSO5054A digital oscilloscope, having an amplitude resolution limit of 2 mV and with activated low-pass-filter limiting the measurements to 25 MHz. The tool exhibited a small DC-offset of 2 mV, which was corrected in Figs. 3 and  4. To improve the readability of the graphs, noise has been reduced by a running average filter, which also slightly reduced the signal amplitudes. For reference, the original data can be found in Supplementary Fig. 8 in the supplementary information.

### Device modeling

Drift-diffusion transport simulations were carried out in Synopsis TCAD. The device structure was setup using process simulations resembling the FDSOI process integration flow. Barrier tunneling was modeled using the WKB approximation for finite-element-method-based simulations. A silicide workfunction of 4.54 eV and a TiN top-gate electrode having a workfunction of 4.65 eV have been used. On-currents have been fitted by the tunneling masses of 0.60 ⋅ *m*_0_ for electrons and holes 0.27 ⋅ *m*_0_, respectively. Band diagrams have been extracted using SVisual.

## Supplementary information

Supplementary Information

## Data Availability

The data that support the plots within this paper and other findings of this study are under restricted access for company policy reason and are available from the corresponding author upon reasonable request.

## Code availability

The computer code used in this study is available from the corresponding author upon reasonable request.
