# Supplementary material for: Three-to-one analog signal modulation with a single back-bias-controlled reconfigurable transistor
Source: Nat Commun. 2022 Nov 17;13:7042. doi: 10.1038/s41467-022-34533-w (PMC9672034; doi:10.1038/s41467-022-34533-w)
Supplement: Supplementary file 1 — Supplementary Information [file 41467_2022_34533_MOESM1_ESM.pdf]

SUPPLEMENTARY INFORMATION

Three-to-One Analog Signal Modulation with a Single Back-Bias-Controlled Reconfigurable Transistor

By

Maik Simon[1], Halid Mulaosmanovic[1,2], Violetta Sessi[2], Maximilian Drescher[2], Niladri Bhattacharjee[1], Stefan Slesazeck[1], Maciej Wiatr[2], Thomas Mikolajick[1,3], and Jens Trommer[1,*]

1 NaMLab gGmbH, 01187 Dresden, Germany

2 Globalfoundries Fab 1, LLC & Co. KG, 01109 Dresden, Germany

3 Chair for Nanoelectronics, TU Dresden, 01062, Dresden, Germany

*Corresponding author: jens.trommer[at]namlab.com

Contents

S0. Qualitative Comparison of different Reconfigurable Field Effect Transistor Variants

S1. Photograph of a 300 mm Wafer used for Back-Bias RFET Integration

S2. Continuously Shift of Id-Vg with applied Back-Bias

S3. Band Diagrams in the Off-State as extracted by TCAD

S4. Remarks on Barrier Extraction Method from TCAD

S5. Frequency Multiplication at 10 KHz Input Frequency

S6. Phase-Shifting at 1 MHz Input Signal Frequency

S7. Frequency-Shift Keying (FSK) and Phase-Shift Keying (PSK)

S8. Three-to-one Signal Modulation Data without Averaging

S0. Qualitative Comparison of different Reconfigurable Field Effect Transistor Variants

Supplementary Table 1. Qualitative comparison of various nanoscale Schottky-barrier-based reconfigurable device variants.

**Device Variant**	**Double-Gate RFET**	**Schottky-Barrier-Bias RFET**		**Back-bias RFET (this work)**
		**Three Top-Gates**	**Front- and Back-Gate**	
**Schematic cross section along channel**	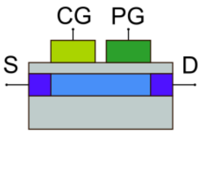	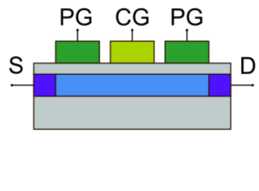	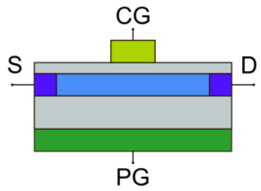	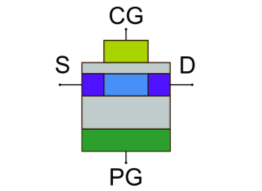
**Transfer curves** for n-mode (blue) as compared to the ambipolar mode of a simple Schottky FET (green). ON and OFF state encircled	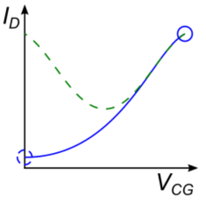	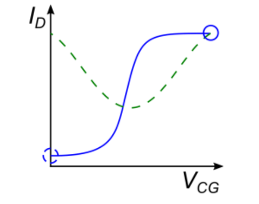	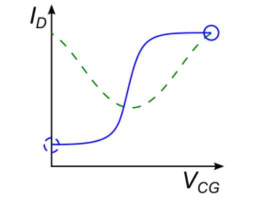	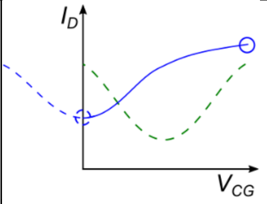
**Schematic of band structure in n-mode** ON- (solid lines) and OFF-state (dashed lines) are shown.	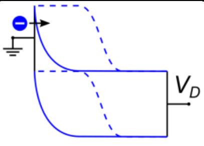	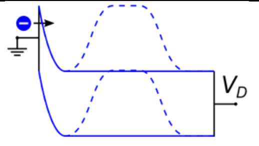	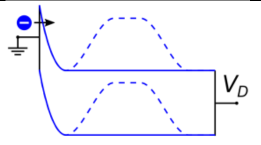	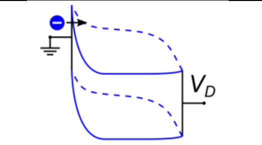
**Doping**	none			
**Operation modes**	2 (n/p)	2 (n/p)	2 (n/p)	3 (n/p/ambipolar)
**Footprint**	large	very large	very large	small
**Program voltage**	low	low	high	high
I_{ON}	comparable			
I_{OFF}	very low	low	low	high
**Subthreshold swing**	moderate	small	small	large
**Threshold voltage**	moderate	low	low	variable

S1. Photograph of a 300 mm Wafer used for Back-Bias RFET Integration

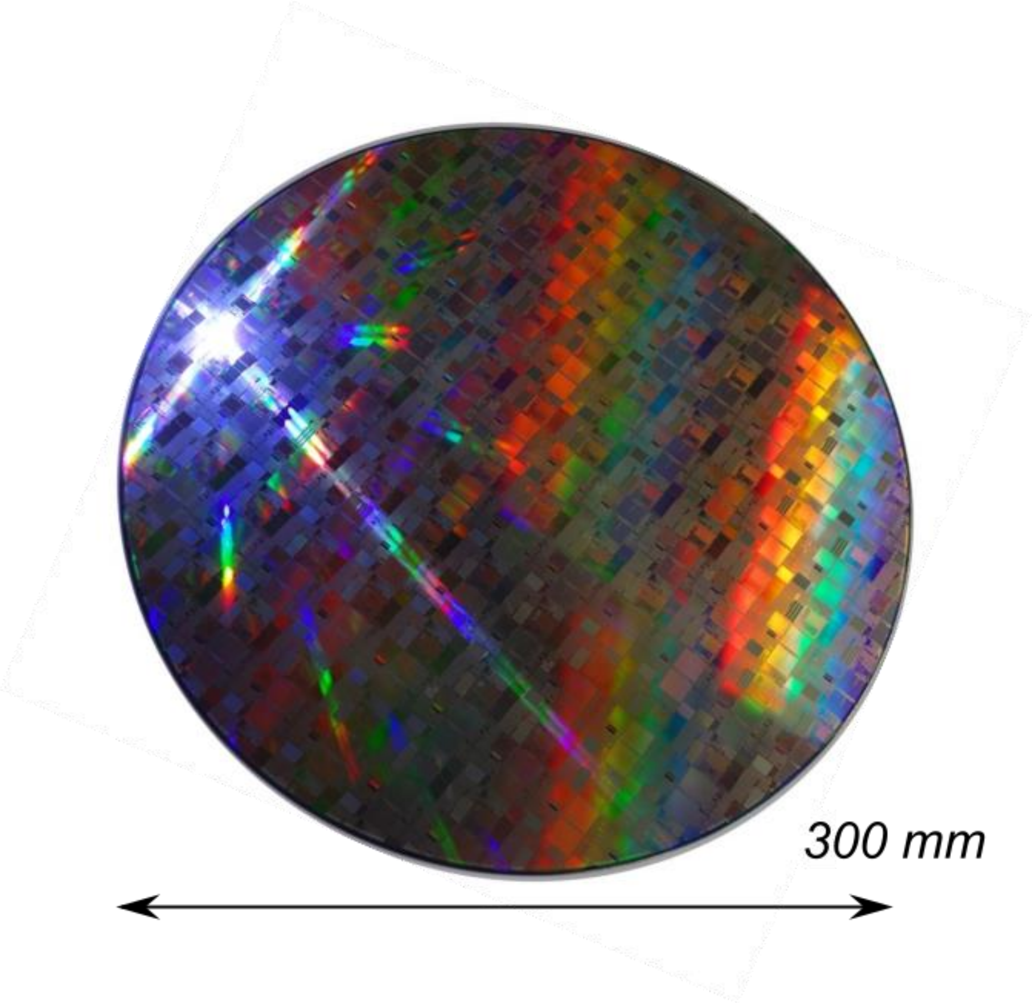

Supplementary Figure 1. Photograph of a 300 mm wafer in 22 nm FDSOI technology with integrated BB-RFET devices.

S2. Continuously Shift of Id-Vg with applied Back-Bias

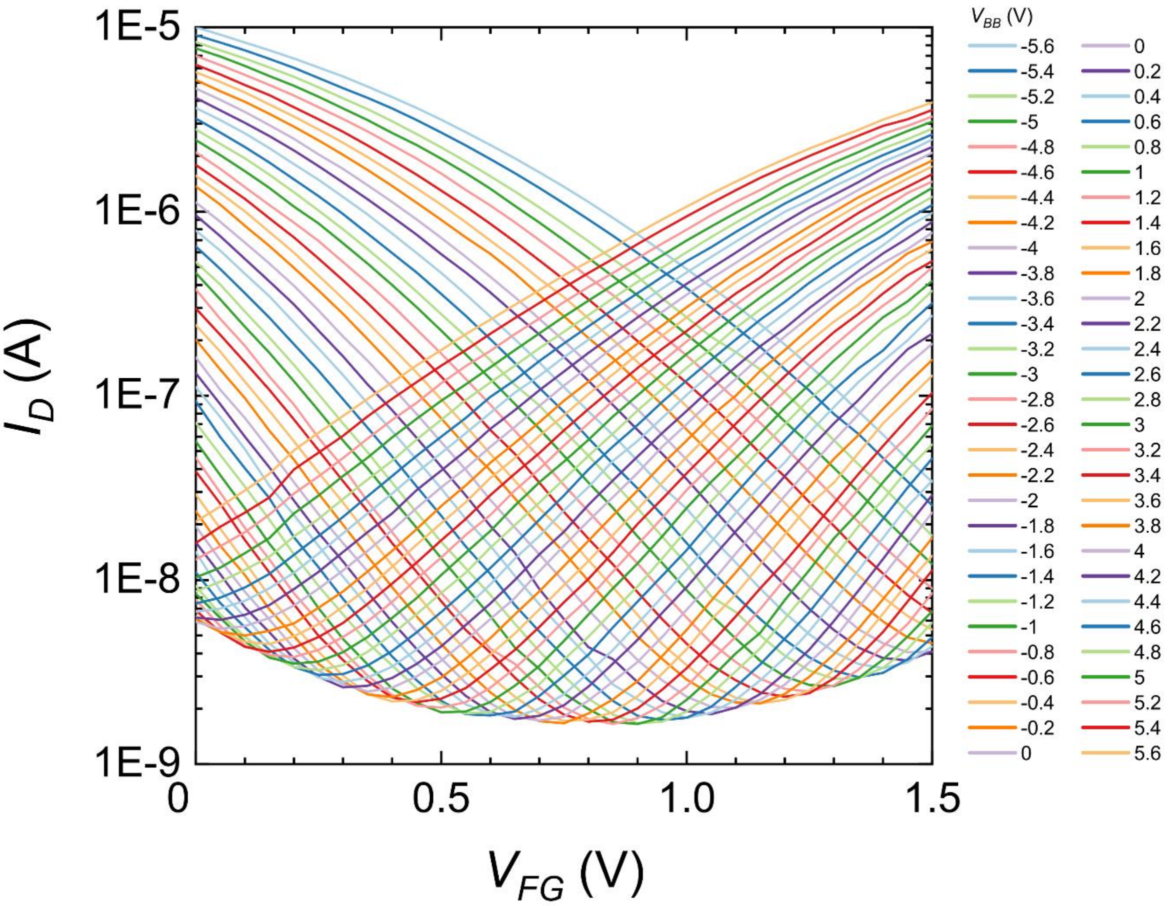

Supplementary Figure 2. Continuous shifting of the transfer characteristics of the device in Figure 1 of the main paper with fine-grain variation of the applied back-bias V_{BB}.

S3. Band Diagrams in the Off-State as extracted by TCAD

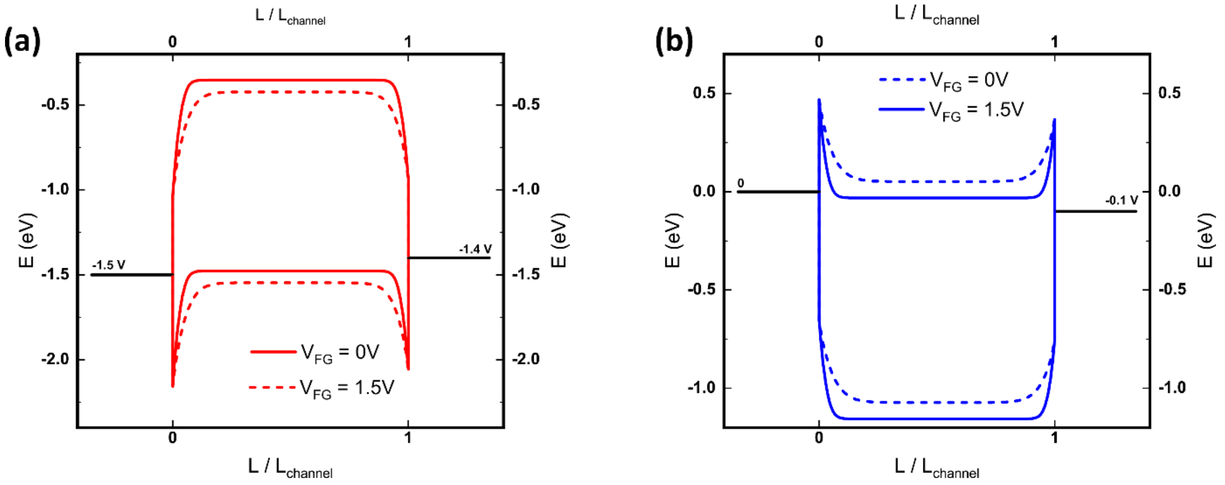

Supplementary Figure 3. Band diagrams extracted from TCAD simulations for a $|V_{DS}|$ of 0.1 V for (a) p-type (red, V_{BB}= -4.0 V) and (b) n-type (blue, V_{BB}= 4.6 V) program, respectively. Potential along the channel is given relative the overall channel length L.

S4. Remarks on Barrier Extraction Method from TCAD

Effective barrier heights under the influence of an external electric field have been determined from TCAD simulations of the individual band structure for each bias condition. The potential of the barrier has been approximated by a triangular shape. A fixed tunneling distance d_{eff} was used as a fitting parameter to yield $\Phi_{B,eff}$ for the different bias conditions. In a simple approximation, the barrier can be seen as permeable (tunneling probability $\Gamma = 1$) above this barrier and opaque (tunneling probability $\Gamma = 0$) below this barrier.

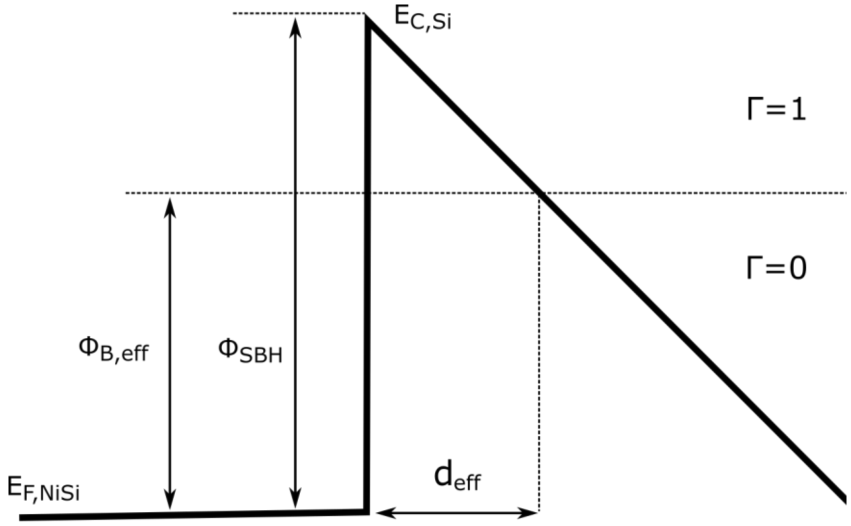

Supplementary Figure 4. Schottky Barrier Model to determine the effective barrier height $\Phi_{B,eff}$ under the influence of an external electric field by using a fixed 'tunneling distance' d_{eff} as a fitting parameter. $E_{F,NiSi}$ is the fermi energy of the silicide contact, $E_{C,Si}$ the conduction band of channel material, and Φ_{SBH} is the natural Schottky barrier height for electrons and Γ the tunneling probability. The height of the barrier under electric field can be approximated by a value $\Phi_{B,eff}$ resulting out of the intersection of the d_{eff} with the triangular barrier.

S5. Frequency Multiplication at 10 KHz Input Frequency

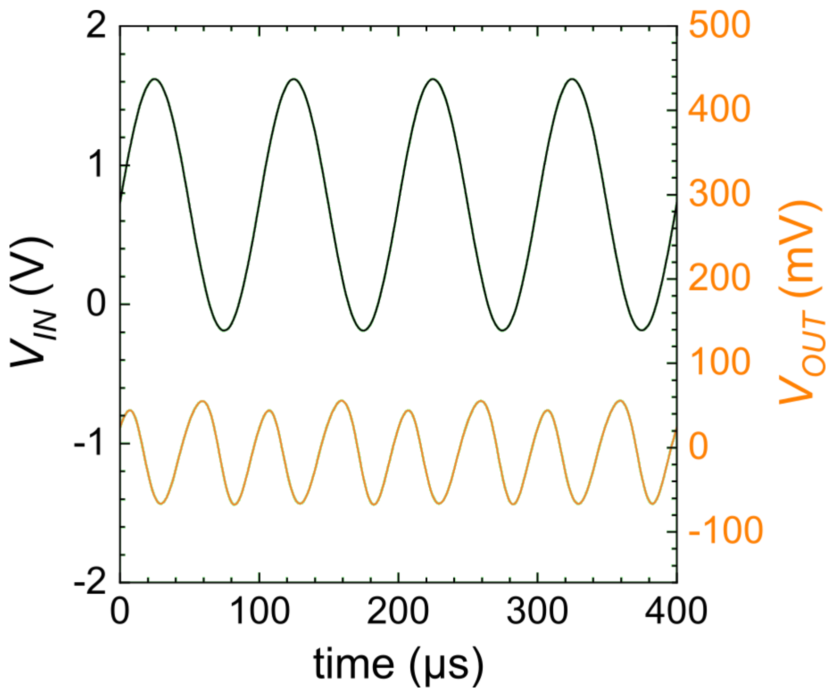

Supplementary Figure 5. Experimental demonstration of frequency doubling exploiting the ambipolar transfer characteristic of the Back-Bias RFET device with an input signal frequency of 10 kHz. Measured input (black, left ordinate) and output (orange, right ordinate) signal of the setup over time.

S6. Phase-Shifting at 1 MHz Input Signal Frequency

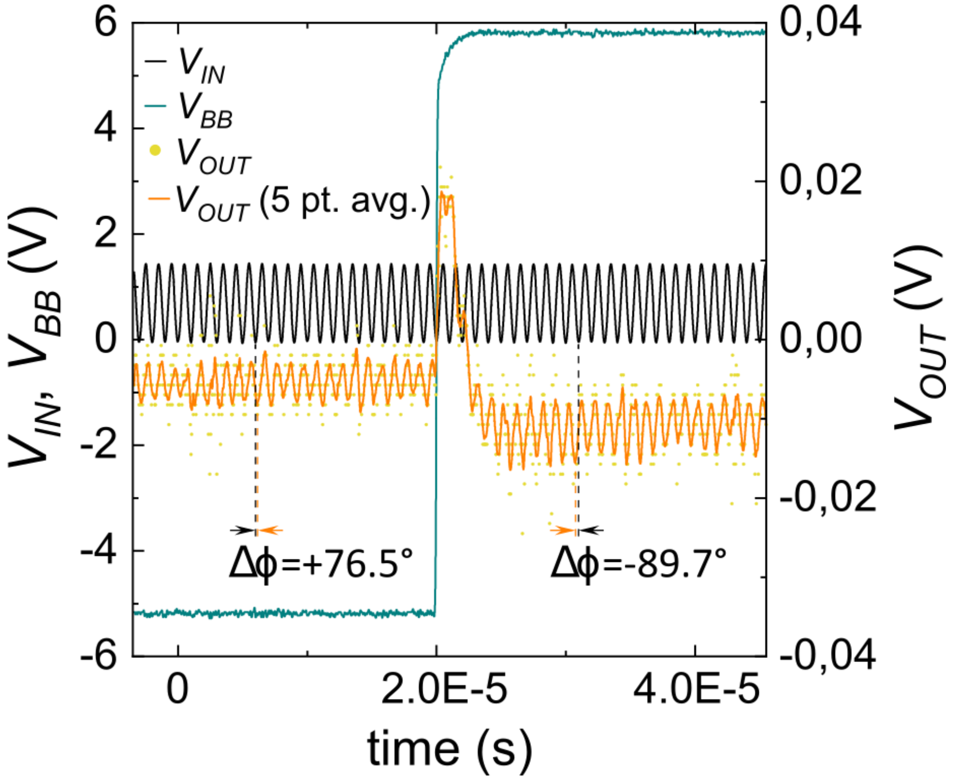

Supplementary Figure 6. Reconfigurable phase shifting by adjusting the applied back-bias (V_{BB}) in a back-bias RFET device with an input signal frequency of 1 MHz. A roughly 180° phase shift is observed in the averaged output data (orange). At this frequency, a considerable amount of noise is present (yellow points) due to the limitations of the setup degrading the quality of the measured signal.

S7. Frequency-shift keying (FSK) and phase-shift keying (PSK)

FSK and PSK are digital modulation schemes, where the modulation of a carrier wave encodes digital data. In the case of FSK the frequency is modulated, whereas for PSK the phase is modulated. Binary PSK is one of the simplest forms, where digital 1s and 0s correspond to two different phases of the modulated signal, e.g. 0° and 180°. Supplementary Fig. 8 illustrates the working principle, where the modulated signal effectively displays phase jumps in correspondence to the change of the binary value. Analog to this behaviour, a binary FSK will modulate the frequency between two distinct states. For example, 100 kHz can represent digital 1s, and 200 kHz represents digital 0s. Both schemes can be nicely implemented with the BB-RFET concept, employing a carrier wave at the front gate and the data input at the back gate. By switching the data input range from [$V_{BB,N}$; GND] to [$V_{BB,N}$; $V_{BB,P}$], the device can be recon-figured between an FSK and PSK mode, respectively.

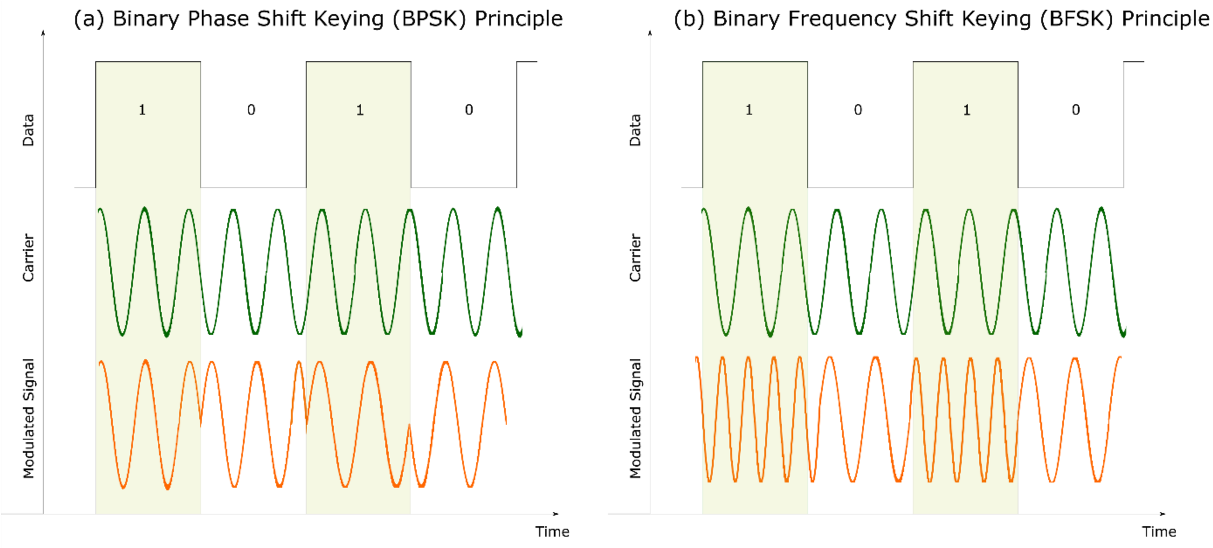

Supplementary Figure 7. Principles of (a) binary phase shift keying and (b) binary frequency shift keying.

S8. Three-to-one Signal Modulation Data without Averaging

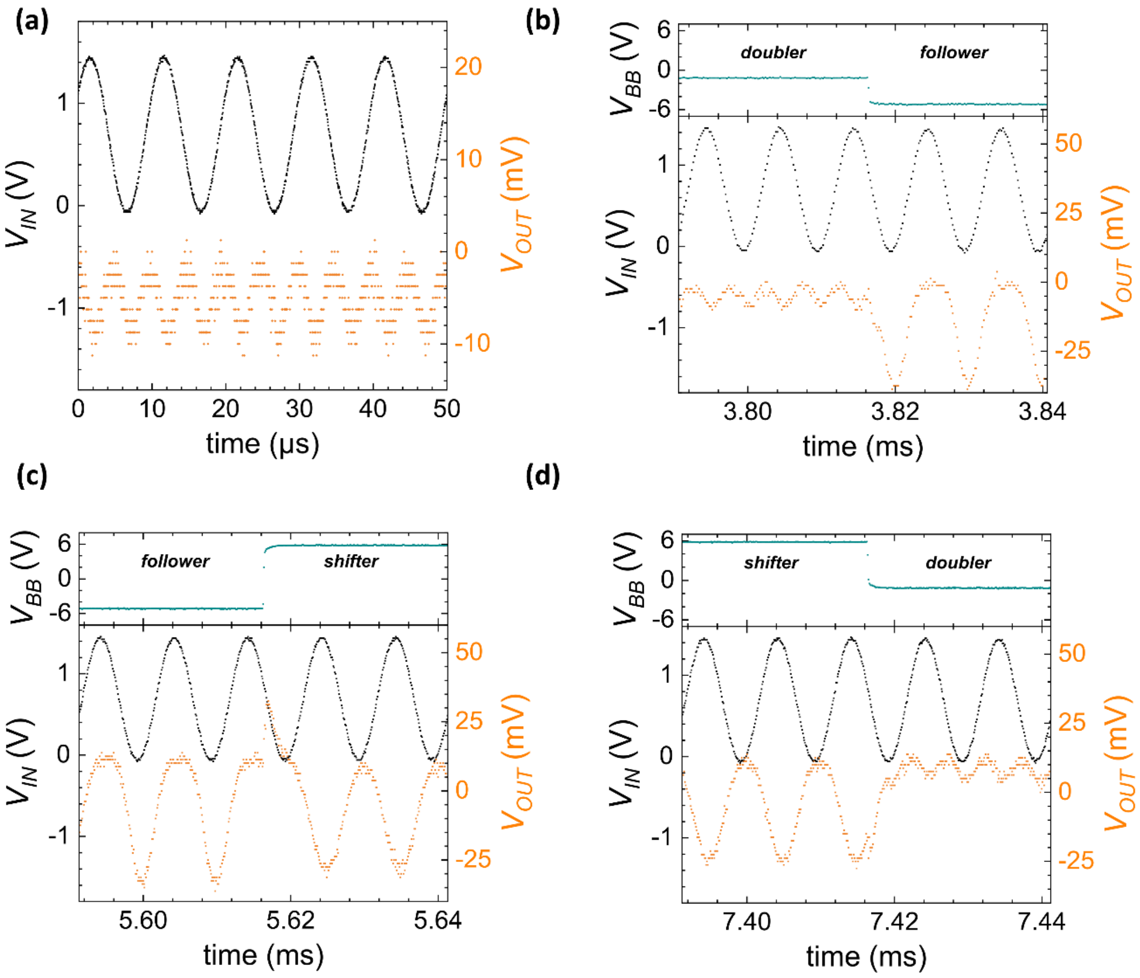

Supplementary Figure 8. Original data for selected operation points of the three-to-one signal modulation measurements without data averaging. (a) frequency doubling operation, (b) frequency doubler to follower transition, (c) follower to shifter transition, and (d) phase shifter to frequency doubler transition.
